# Factors influencing clinical pregnancy outcome of in vitro fertilization/intracytoplasmic sperm injection in older women

**DOI:** 10.4314/ahs.v23i2.73

**Published:** 2023-06

**Authors:** Jinyu Gao, Zhaohua Liu, Yao Zhong, Nannan Li, Tingting Tang

**Affiliations:** Reproductive Medicine Center, Hunan Provincial Maternal and Child Health Care Hospital, Hu'nan Province, China

**Keywords:** Older pregnant woman, in vitro fertilization/intracytoplasmic sperm injection, pregnancy outcome, risk factor, nomogram prediction model

## Abstract

**Objective:**

To analyse the factors influencing clinical pregnancy outcome of in vitro fertilization/intracytoplasmic sperm injection (IVF/ICSI) in older women, and to establish a risk prediction model.

**Methods:**

A total of 425 patients receiving IVF/ICSI from March 2018 to March 2020 were divided into pregnancy group (n=194) and non-pregnancy group (n=231). The factors affecting the outcomes of IVF/ICSI were explored by univariate and multivariate logistic regression analyses. A nomogram prediction model was constructed.

**Results:**

The two groups had significantly different age, body mass index, dysmenorrhea, parity, times of full-term births, history of cesarean section, basal follicle stimulating hormone, basal antral follicle count (AFC), number of high-quality embryos, and basal estradiol, luteinizing hormone and endometrial thickness on the day of human chorionic gonadotropin (HCG) administration (P<0.05). Age ≥40 years old, dysmenorrhea, history of cesarean section, basal AFC<9, number of high-quality embryos <4, and endometrial thickness on the day of HCG administration <11 mm led to IVF/ICSI failure. The established model exhibited high calibration and discrimination degrees in predicting the outcome of IVF/ICSI.

**Conclusion:**

The risk prediction model for the pregnancy outcome of IVF/ICSI in older women helps evaluate the fertility probability and risk, providing references for formulating reasonable assisted reproduction plans.

## Introduction

As the society develops, more women have chosen to marry and to give birth late^1^. However, older pregnant women are usually in a state of decreased ovarian reserve function, and the incidence rate of infertility rises with aging^2^. The incidence rate of infertility in women aged 35-39 years old is 30%, while that in women aged 39-44 years old increases to 64%^3^. The success rate of natural pregnancy greatly reduces in older pregnant women, so assisted reproductive technology is needed to meet their fertility demand^4^. In vitro fertilization/intracytoplasmic sperm injection (IVF/ICSI) is an important method for treating infertility^5^. The proportion of older pregnant women hoping to get pregnant through IVF/ICSI is gradually increasing. However, the clinical pregnancy outcome is still unsatisfactory^6^. In the process of assisted reproduction, older pregnant women have small numbers of retrieved oocytes and transferable embryos, poor quality of transferable embryos, low clinical pregnancy rate and high abortion rate, resulting in the failure of operation^7^. The embryo implantation rate drops gradually in older pregnant women at a speed of 2.7% per year^8^. In the field of reproductive medicine, therefore, elevating the clinical pregnancy success rate of older pregnant women receiving IVF/ICSI remains rather difficult and thus needs extensive research^9^.

So far, the factors leading to IVF/ICSI failure in older pregnant women have not been fully elucidated. Thereby motivated, the factors affecting the clinical pregnancy outcome of IVF/ICSI in older women were investigated herein, aiming to provide valuable references for formulating assisted reproduction plans for older pregnant women and to increase the clinical pregnancy success rate.

## Materials and methods

### General data

A total of 425 patients receiving IVF/ICSI in our hospital from March 2018 to March 2020 were divided into pregnancy group (n=194) and non-pregnancy group (n=231). In the pregnancy group, the women were aged 36-43 years old, with an average age of (39.18±1.85) years old. In the non-pregnancy group, the women were aged 37-45 years old, with a mean of (40.64 ±2.12) years old. This study was reviewed and approved by the medical ethics committee of our hospital.

The inclusion criteria involved: (1) patients meeting the relevant ethical principles in the Code of Human assisted Reproductive Technology^10^, (2) those aged >35 years old, (3) those meeting the relevant indications of IVF/ICSI, (4) those receiving fresh cycle embryo transfer (ET), and (5) those who and whose family members were informed of this study.

The exclusion criteria were as follows: (1) those who or whose partners suffered from sexually transmitted diseases, acute infections of the genitourinary system or severe mental diseases, (2) those who or whose partners had serious bad habits such as taking drugs, (3) those with abnormal development of the reproductive tract or uterus, (4) those suffering from infertile diseases or hereditary diseases stipulated in the Mother and Infant Health Care Law of the People's Republic of China, or (5) those who or whose partners were exposed to teratogenic drugs or poisons or rays and in the active period.

### Collection of general data

The general data of patients were collected through electronic medical records, including age, height, weight, body mass index (BMI), duration of infertility, type of infertility (primary or secondary infertility), age of menarche, dysmenorrhea, parity, times of full-term births, history of cesarean section, basal follicle stimulating hormone (FSH), basal luteinizing hormone (LH), basal estradiol (E_2_), basal testosterone (T), and basal antral follicle count (AFC).

### Observation indices

The insemination method (IVF or ICSI), numbers of oocytes retrieved, high-quality oocytes, high-quality embryos and transferred embryos, E_2_, LH, progesterone (P), endometrial thickness on the day of human chorionic gonadotrophin (HCG) administration, and application duration and total dosage of gonadotropin (Gn) were recorded.

Evaluation criteria for embryo quality^11^: (1) The quality of embryos at cleavage stage was assessed and classified into 4 grades according to the number, uniformity and proportion of blastomeres: Grade I: Blastomeres in a uniform size, with no obvious DNA fragments. Grade II: Blastomeres in a slightly uniform size, with a DNA fragmentation rate <20%. Grade III: Blastomeres in an obviously non-uniform size, with a DNA fragmentation rate of 20-50%. Grade IV: A DNA fragmentation rate >50%. Embryos with double pronuclei at 1 d after fertilization and 7-9 cells at 3 d after fertilization and at morphological grade I-II were defined as high-quality embryos at cleavage stage. (2) The quality of embryos at blastocyst stage was evaluated and divided into 6 phases according to the size of blastocyst cavity and the degree of hatching. Among them, blastocysts at >phase 3 were scored according to inner cell mass and trophoblast cells, which were divided into grades A, B and C. Embryos with blastocyst morphological score ≥3 BB (i.e. the size and hatching degree of blastocyst cavity were at phase 3, and the inner cell mass and trophoblast cells were scored grade B) at 5 d after fertilization and blastocyst morphological score ≥4 BB 6 d after fertilization were defined as high-quality embryos at the blastocyst stage.

Clinical pregnancy was determined as follows: The level of serum β-HCG was detected 14 d after ET, and the patients with positive results were examined by ultrasound 28-35 d after ET. Clinical pregnancy was confirmed in patients with gestational sac and fetal heart and fetal buds in the gestational sac.

### Construction and validation of nomogram prediction model

Multivariate logistic regression analysis was used to analyse the factors affecting the clinical pregnancy outcome of IVF/ICSI in older women, and the variables with significant differences were assigned. The 425 older pregnant women were randomly divided into training set (n=340) and validation set (n=85) at a ratio of 4:1. The training set was used to construct the nomogram prediction model, while the validation set was utilized for the internal validation of the nomogram prediction model. The nomogram prediction model was established using R software (R 3.3.2) and the rms software package. The calibration of the nomogram prediction model was evaluated using the calibration curve, and the discrimination of the nomogram prediction model was evaluated by the receiver operating characteristic (ROC) curve.

### Statistical analysis

SPSS19.0 software was utilized for statistical analysis, and GraphPad Prism 5.0 software was employed for plotting. The numerical data were expressed as percentage, and chi-square test was performed for intergroup comparison. The measurement data were expressed as mean ± standard deviation, and independent t-test was conducted for intergroup comparison. P<0.05 indicated that difference was statistically significant.

## Results

### General data and IVF/ICSI status

Significant differences were observed in age, BMI, dysmenorrhea, parity, times of full-term births, history of cesarean section, basal FSH and basal AFC between the two groups (P<0.05). Nevertheless, there were no significant differences in other general data between the two groups (P>0.05) (Table 1).

**Table 1:** General data

Group	Pregnancy group (n=194)	Non-pregnancy group (n=231)	*t*/χ^2^	P
Age (year)	39.18±1.85	40.64±2.12	7.491	0.000
BMI (kg/m^2^)	23.25±2.64	22.34±2.58	3.584	0.000
Duration of infertility (year)	3.34±2.02	3.05±1.95	1.502	0.134
Type of infertility [n (%)]			0.236	0.627
Primary infertility	31 (15.98)	33 (14.29)	/	/
Secondary infertility	163 (84.02)	198 (85.71)	/	/
Age of menarche (year)	13.61±1.68	13.48±1.47	0.851	0.395
Dysmenorrhea [n (%)]			5.596	0.018
Yes	3 (1.55)	14 (6.06)	/	/
No	191 (98.45)	217 (93.94)	/	/
Parity (time)	0.60±0.52	0.73±0.53	2.540	0.011
Times of full-term births (time)	0.53±0.49	0.66±0.50	2.694	0.007
History of cesarean section [n (%)]			9.582	0.002
Yes	19 (9.79)	48 (20.78)	/	/
No	175 (90.21)	183 (79.22)	/	/
Basal FSH (U/L)	7.82±3.21	9.45±4.06	4.528	0.000
Basal LH (U/L)	4.85±2.13	4.64±2.08	1.025	0.306
Basal E_2_ (pmol/L)	206.73±51.64	212.85±53.49	1.194	0.233
Basal T (nmol/L)	1.11±0.72	1.05±0.69	0.875	0.382
Basal AFC	10.13±4.72	7.84±4.15	5.321	0.000

### IVF/ICSI status

Significant differences were found in the number of high-quality embryos, and E2, LH and endometrial thickness on the day of HCG administration between the two groups (P<0.05). However, no significant differences were observed in the insemination method, number of oocytes retrieved, high-quality oocytes, transferred embryos, P on the day of HCG administration, and application duration and total dosage of Gn between the two groups (P>0.05) (Table 2).

**Table 2:** IVF/ICSI status

Group	Pregnancy group (n=194)	Non-pregnancy group (n=231)	*t*	P
Insemination method [n (%)]			0.717	0.397
IVF	160 (82.47)	183 (79.22)	/	/
ICSI	34 (17.53)	48 (20.78)	/	/
Number of oocytes retrieved	7.47±3.65	6.98±3.42	1.427	0.154
Number of high-quality oocytes	6.39±3.48	5.78±3.12	1.904	0.058
Number of high-quality embryos	4.48±2.23	3.74±2.08	3.535	0.000
Number of transferred embryos	1.86±0.31	1.90±0.29	1.372	0.171
E_2_ on the day of HCG administration (nmol/L)	126.37±27.31	110.26±25.83	6.239	0.000
LH on the day of HCG administration (U/L)	2.13±1.05	2.90±1.28	6.697	0.000
P on the day of HCG administration (nmol/L)	3.85±1.12	3.96±1.23	0.956	0.339
Endometrial thickness on the day of HCG administration (mm)	11.53±2.42	10.73±2.38	3.425	0.001
Application duration of Gn (d)	10.12±2.31	9.82±2.06	1.415	0.158
Total dosage of Gn (IU)	2347.65±683.74	2328.45±675.82	0.290	0.772

### Univariate and multivariate logistic regression analysis results of factors affecting pregnancy outcome

Age ≥40 years old odds ratio (OR): 3.876, 95% confidence interval (CI): 3.023-4.729, P=0.005), dysmenorrhea (OR: 3.586, 95% CI: 2.736-4.436, P=0.012), history of cesarean section (OR: 3.648, 95% CI: 2.837-4.459, P=0.010), basal AFC <9 (OR: 4.0758, 95% CI: 2.9875.169, P=0.001), number of high-quality embryos <4 (OR: 4.876, 95% CI: 4.023-5.729, P<0.001) and endometrial thickness on the day of HCG administration <11 mm (OR: 4.583, 95% CI: 3.674-5.519, P<0.001) were associated with the clinical pregnancy outcome of IVF/ICSI in older women (Table 3).

**Table 3:** Univariate logistic regression analysis results of factors affecting pregnancy outcome

Item	β	S.E.	Wald χ^2^	P	OR (95% CI)
Age ≥40 years old	1.731	1.239	3.859	0.005	3.876 (3.023~4.729)
Dysmenorrhea	1.648	1.421	5.438	0.012	3.586 (2.736~4.436)
History of cesarean section	1.259	1.764	3.462	0.010	3.648 (2.837~4.459)
Basal AFC <9	1.583	1.587	4.231	0.001	4.078 (2.987~5.169)
Number of high-quality embryos <4	1.374	1.321	3.745	0.000	4.876 (4.023~5.729)
Endometrial thickness on the day of HCG administration <11 mm	1.682	1.425	2.647	0.000	4.583 (3.674~5.519)

**Figure 1:**
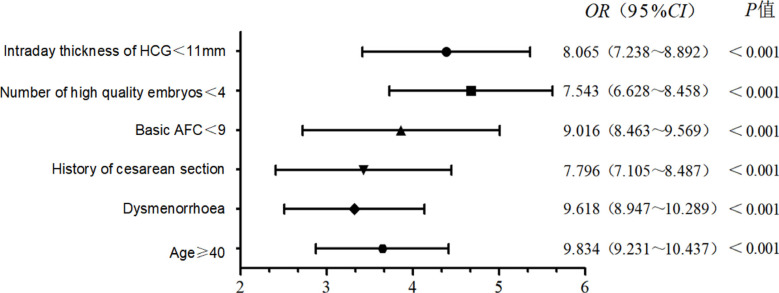
Multivariate logistic regression analysis results of factors affecting pregnancy outcome

Using the indices associated with the clinical pregnancy outcome of IVF/ICSI in older women in univariate analysis as independent variables, and the pregnancy outcome (pregnancy=0 and non-pregnancy=1) as the dependent variable, multivariate logistic regression analysis was employed to analyse the factors affecting the pregnancy outcome. Age ≥40 years old, dysmenorrhea, history of cesarean section, basal AFC <9, number of high-quality embryos <4, and endometrial thickness on the day of HCG administration <11 mm were all risk factors for the clinical pregnancy failure of IVF/ICSI in older pregnant women (Figure 1).

### Construction and evaluation of nomogram prediction model

**Figure 2:**
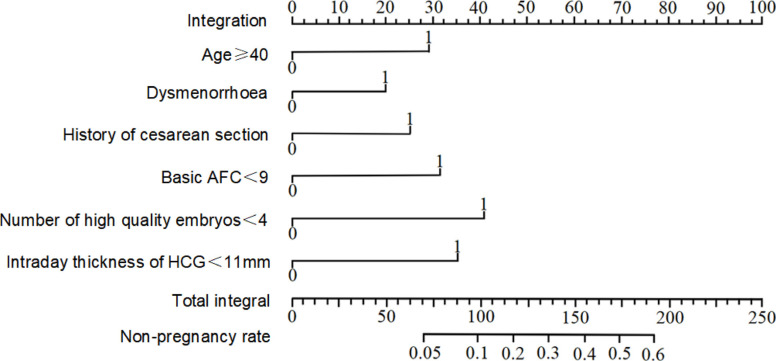
Construction of nomogram prediction model

According to the results of multivariate regression analysis, a nomogram model was constructed to predict the clinical pregnancy failure of IVF/ICSI in older pregnant women. The scores for age ≥40 years old, dysmenorrhea, history of cesarean section, basal AFC <9, number of high-quality embryos <4, and endometrial thickness on the day of HCG administration <11 mm was 20, 25, 32, 42 and 35 points, respectively. The total score was 181 points, based on which the corresponding clinical pregnancy failure rate of IVF/ICSI in older pregnant women was 54.35% (Figure 2).

**Figure 3:**
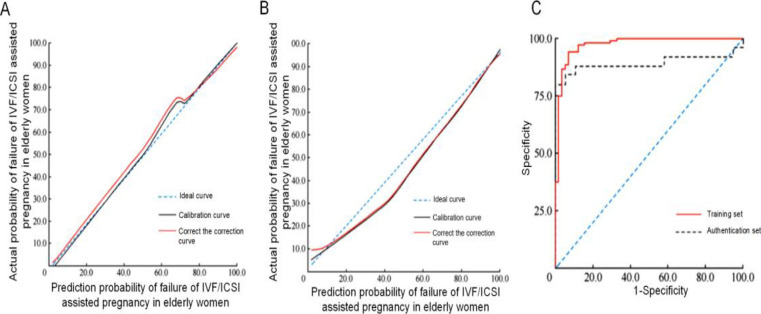
Evaluation of nomogram prediction model. A: Calibration curve in the nomogram prediction model of training set, B: Calibration curve in the nomogram prediction model of validation set, C: ROC curves of training set and validation set

In the calibration curve of the training set, when the predicted probability was <50%, the difference between actual and predicted probabilities was small. When the predicted probability was 50-70%, the actual probability was slightly lower than the predicted one. When the predicted probability was >70%, the calibration curve did not deviate from the ideal one, showing a high degree of calibration. In the calibration curve of the validation set, the calibration curve and corrected one deviated from the ideal curve. When the predicted probability was 40%, the difference between actual and predicted probabilities was the largest (10%). The areas under the ROC curves predicted by the nomogram prediction model of training and validation sets were 0.926 (95% CI: 0.885-0.967, P<0.001) and 0.884 (95% CI: 0.852-0.916, P<0.001), respectively. The sensitivities were 91.64% and 82.73%, and the specificities was 94.25% and 88.72%, respectively, showing high a degree of discrimination (Figure 3).

## Discussion

Due to the rapid development of society, the infertility rate of women has increased annually_12_. After the age of 35, the fertility of women declines yearly with aging, and the pregnancy rate of 45-year-old women is only 10%^13^. IVF/ICSI has become a crucial assisted reproduction method^14^. Nevertheless, the success rate is still far lower than the normal pregnancy rate in healthy people. In particular, the success rate of assisted reproduction remarkably decreases in older pregnant women^15^. The clinical pregnancy success rate and live birth rate of IVF/ICSI in women over 40 years old are obviously lower than those of young infertile patients^16^.

Lu et al. explored the factors affecting the clinical pregnancy outcome of female long-term IVF/ICSI, and found that older age was the risk factor leading to the failure of assisted reproduction in older pregnant women^17^. The number, quality and ovarian reserve function of oocytes decrease with increasing female age, and the response capability of ovary to ovulation induction drugs declines, so both oocyte retrieval rate and transferable embryos reduce, thus lowering the success rate of IVF/ICSI. Pelvic endometriosis, as a common cause of dysmenorrhea in women, has an incidence rate of up to 35-50% in infertility patients with dysmenorrhea. Endometriosis leads to poor oocyte quality, ovulation disorder, tubal adhesion and poor endometrial capacitance, thereby increasing the failure rate of IVF/ICSI in patients with infertility and dysmenorrhea. In this study, history of cesarean section was a factor leading to the failure of IVF/ICSI in older pregnant women. Women undergoing cesarean section have the risks such as scar diverticulum, scar pregnancy and uterine rupture. Patounakis et al. found that history of cesarean section increased the difficulty of ET, and the average time of ET was prolonged by 30 s^18^. Zhang et al. revealed that basal AFC was a factor influencing the clinical pregnancy outcome of fresh cycle IVF-ET in older women^19^. Basal AFC, which is a vital index to reflect the ovarian reserve function, has high predictive value for ovarian response. Palmsten et al. found that the pregnancy rate in women ≥38 years old receiving IVF-ET was dramatically lower than that of women <38 years old, the number of high-quality embryos was evidently smaller than that of women <38 years old, and the number of high-quality embryos was related to the pregnancy rate of older pregnant women^20^. Moreover, Chen et al. reported that endometrial thickness on the day of HCG administration was a factor affecting the clinical pregnancy outcome of female IVF-ET/ICSI^21^. Endometrial thickness can reflect the endometrial receptivity which is an important factor influencing the pregnancy outcome.

In this study, age ≥40 years old, dysmenorrhea, history of cesarean section, basal AFC <9, number of high-quality embryos <4, and endometrial thickness on the day of HCG administration <11 mm were all risk factors leading to the clinical pregnancy failure of IVF/ICSI in older pregnant women. The nomogram model for predicting the pregnancy outcome of assisted reproduction in older pregnant women had high degrees of calibration and discrimination, showing a high predictive value. In clinical practice, attention should be paid to the above risk factors. Older pregnant women should receive assisted reproduction treatment as soon as possible, and reasonable assisted reproduction plans should be formulated to raise the clinical pregnancy rate.

In conclusion, analysing factors affecting the clinical pregnancy outcome of IVF/ICSI in older women and constructing a risk prediction model are conducive to the evaluation of patients' fertility probability and risk, providing references for formulating reasonable assisted reproduction plans for older pregnant women.
